# How do we prevent scientific fraud?

**DOI:** 10.1093/braincomms/fcac217

**Published:** 2022-09-06

**Authors:** Tara L Spires-Jones

**Affiliations:** Edinburgh, UK

## Abstract

Our editor discusses scientific fraud and ways we can discourage it.

## Graphical Abstract

**Graphical abstract:**
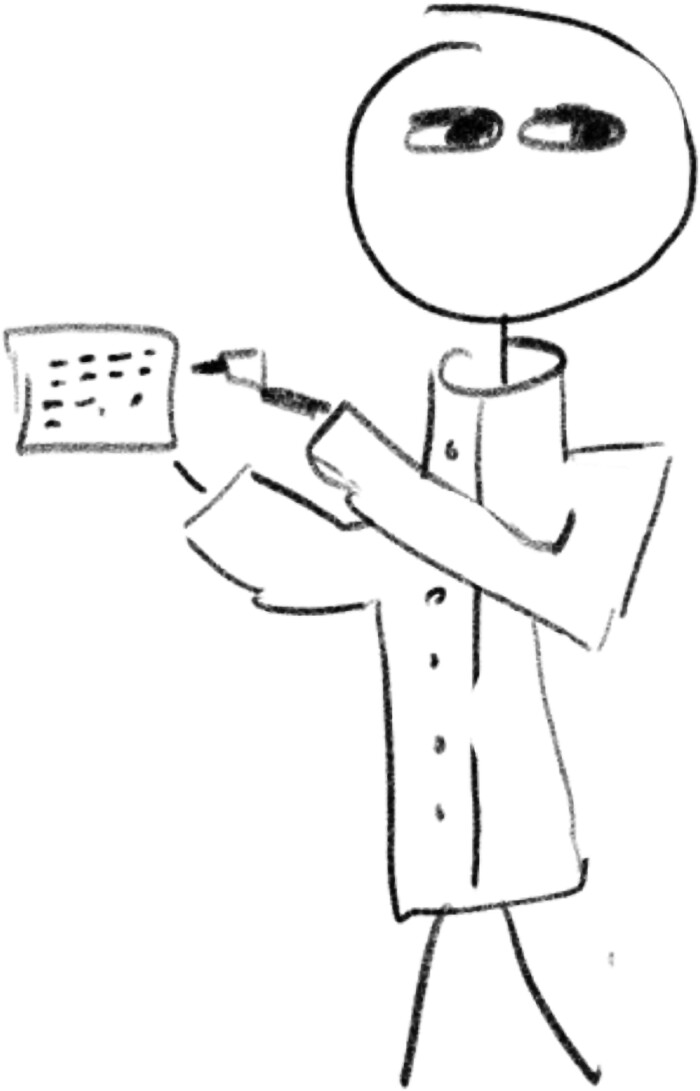

Welcome to Volume 4 Issue 5 of *Brain Communications*. You may have heard recently of a high-profile case of alleged scientific fraud in the Alzheimer’s disease field.^1^ The first author of a *Nature* paper describing a dodecameric form of oligomeric amyloid beta has been accused of manipulating bands on Western blots, one of the key pieces of evidence in the paper. There was a storm in the media and within the scientific community including the *Science*^1^ report and a large conversation on Alzform (https://www.alzforum.org/news/community-news/sylvain-lesne-who-found-av56-accused-image-manipulation). Concerns were raised that the billions of pounds invested in amyloid-directed therapeutics were wasted. This shows how damaging isolated cases of fraud can be to the reputation of the scientific community. Luckily, this case of alleged fraud is not nearly as damaging as the scandal linking the measles, mumps, and rubella vaccine to autism risk which was based on falsified data on a case series of 12 children.^2^ That case of fraud led to people dying because of fear of a safe vaccine.

My take on the oligomeric amyloid dodecamer fraud allegations is that it is actually an illustration of the scientific process working towards an accurate picture of the world. Since other scientists have not been able to replicate the dodecamer data, that particular line of research has fizzled out in favour of more replicable work, such as the synaptic toxicity of soluble amyloid beta and its effects on microglia and astrocytes. The dodecamer paper was by no means the foundation of the amyloid hypothesis, which emerged from genetic studies linking mutations that increase amyloid beta to familial Alzheimer’s disease. And of course, the Alzheimer’s field extends far beyond amyloid studies with significant investments in researching tau, glia, etc.

Scientists are only human and there will be some bad apples, but I think we can do more as a field to stop fraud in neuroscience. As scientists, we face pressure to publish novel, positive results to get the next grant, the next promotion, and to keep the people who work for us employed and paying their bills. Luckily, scientific fraud is rare, but it would be even rarer if our ecosystem rewarded rigorous science over novelty. There are many initiatives in this direction including grant panel members being told not to consider the impact factor of journals as a mark of quality, and outstanding work by societies, such as the British Neuroscience Association, promoting credibility in neuroscience (https://www.bnacredibility.org.uk/). The publishing industry is starting to play its part as well, through movement towards open access and open data.

One of our goals at *Brain Communications* is to be a force for good in the field by publishing rigorous, reproducible papers. I hope we are doing our part to help the field. We have already published excellent work along these lines, for example, a study replicating blood biomarkers of dementia severity in a different cohort,^3^ using data from eight cohorts to develop reference values for plasma neurofilament light chain in healthy people,^4^ and contradictory findings in a rat model of Parkinson’s disease.^5,6^ Please continue to send us your work including negative data, replication studies, and registered reports. And, if you see a paper you disagree with in our journal, please get in touch and write a letter to the Editor to keep the discussion going.

The cover image for this issue comes from He *et al.*^7^ and shows an overview of the cholinergic projections from the lateral parabrachial nucleus to the central nucleus of amygdala in wild-type mice by fluorescent micro-optical sectioning tomography.
